# Effects of nanopillars and surface coating on dynamic traction force

**DOI:** 10.1038/s41378-022-00473-0

**Published:** 2023-01-05

**Authors:** Yijun Cheng, Stella W. Pang

**Affiliations:** 1 Department of Electrical Engineering, City University of Hong Kong, Kowloon, Hong Kong, China; 2 Centre for Biosystems, Neuroscience, and Nanotechnology, City University of Hong Kong, Kowloon, Hong Kong, China

**Keywords:** Nanoscale devices, Other nanotechnology

## Abstract

The extracellular matrix serves as structural support for cells and provides biophysical and biochemical cues for cell migration. Topography, material, and surface energy can regulate cell migration behaviors. Here, the responses of MC3T3-E1 cells, including migration speed, morphology, and spreading on various platform surfaces, were investigated. Polydimethylsiloxane (PDMS) micropost sensing platforms with nanopillars, silicon oxide, and titanium oxide on top of the microposts were fabricated, and the dynamic cell traction force during migration was monitored. The relationships between various platform surfaces, migration behaviors, and cell traction forces were studied. Compared with the flat PDMS surface, cells on silicon oxide and titanium oxide surfaces showed reduced mobility and less elongation. On the other hand, cells on the nanopillar surface showed more elongation and a higher migration speed than cells on silicon oxide and titanium oxide surfaces. MC3T3-E1 cells on microposts with nanopillars exerted a larger traction force than those on flat PDMS microposts and had more filopodia and long protrusions. Understanding the relationships between platform surface condition, migration behavior, and cell traction force can potentially lead to better control of cell migration in biomaterials capable of promoting tissue repair and regeneration.

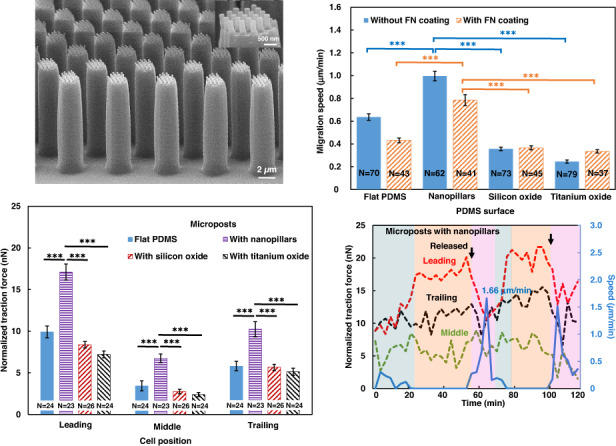

## Introduction

The extracellular matrix (ECM) microenvironment provides biophysical and biochemical cues such as topography, stiffness, functional groups, and surface energy for cells^1,2^. These cues can regulate cell proliferation, apoptosis, migration, differentiation, and spreading^3^. Furthermore, cell migration is an essential physiological and pathological process related to embryo development, wound healing, tumor metastasis, and angiogenesis^4–6^. For example, osteoblasts (e.g., MC3T3-E1 cells) undergo cell migration to form calcified bone matrix, which is essential for the precise coordination of bone remodeling^7^. Therefore, it is useful to develop artificial ECM with proper topography and surface coating to control cell migration for potential application in regenerative medicine. These artificial ECMs could be used to replace damaged tissues or organs^8^. They can also be applied to study cell biomechanics, which could influence various pathological processes^9^.

Researchers have previously shown that the topography and surface chemistry of biomaterials could influence cell migration^10–12^. Platforms with designed topographical features were developed to guide cell migration^13–15^. Mouse osteoblast cells were guided to migrate along gratings of specific width and height^16^. In addition, by introducing asymmetrical topography, cells could migrate with better directionality^14^. Surface roughness is another essential factor affecting cell migration behaviors^17^. Nanoscale roughness has been found to positively affect cell adhesion, growth, and migration^18^. Compared with flat surfaces or gratings, platforms with nanoholes or nanopillars could promote the formation of filopodia and long protrusions, thus increasing the migration speed of MC3T3-E1 cells^19^.

In addition, the surface chemistry of biomaterials, such as wettability, is known to influence cell responses. Hydrophilic silicon oxide and titanium oxide are often used as coatings for implanted devices due to their good biocompatibility^20,21^. Surface energy of the ECM can control fluid interactions, cell adhesion, and protein adsorption, thus influencing tissue formation^22^. It has been reported that surfaces with a contact angle of ~70° provide the best control of cell behaviors^23^. Cell adhesion was the highest for contact angles between 60° and 80° and decreased for larger contact angles. In this work, nanopillars and surface coatings were used to change the surface energy of platforms, which provided a broader understanding of the interactions between cells and platforms with various surface conditions.

Cells sense the biophysical and biochemical properties of the surface to guide subsequent cellular responses^24^. When cells make contact with a surface, cell traction force is generated by actomyosin interaction and actin polymerization^25^. For example, cells could respond to substrate stiffness through the transmission of cell traction force that affects cell morphology and migration^26,27^. Furthermore, topographical guidance and confinement have been shown to influence the cell traction force^28,29^. In addition, nanopillars have been shown to change cell migration by influencing cell adhesion, the cytoskeleton, and the traction force acting on the surface^30^. Therefore, monitoring the cell traction force on nanopillars is critical in understanding cell-ECM interactions. Using microposts as sensors to monitor the traction force distribution over time on different surfaces is useful for designing biomaterials to better control cell migration behaviors.

In this work, cell migration behaviors were studied systematically on different surfaces, including surfaces with flat PDMS, nanopillars, silicon oxide, and titanium oxide. Subsequently, PDMS micropost sensing arrays with nanopillars and various oxide coatings on top were developed to track the cell traction force of MC3T3-E1 cells. The dynamic traction force for cells migrating on different surfaces was measured and analyzed. Cells on microposts with nanopillars were found to exert a larger traction force than on flat PDMS microposts or with oxide coatings. More filopodia and long protrusions were observed on microposts with nanopillars. This comprehensive study of the relationships between surface conditions, migration behavior, and cell traction force could help to engineer an ECM with proper surface conditions for controlling cell migration to promote tissue repair and regeneration.

## Experiment and methods

### Fabrication technology for micropost sensing platforms

**Fig. 1:**
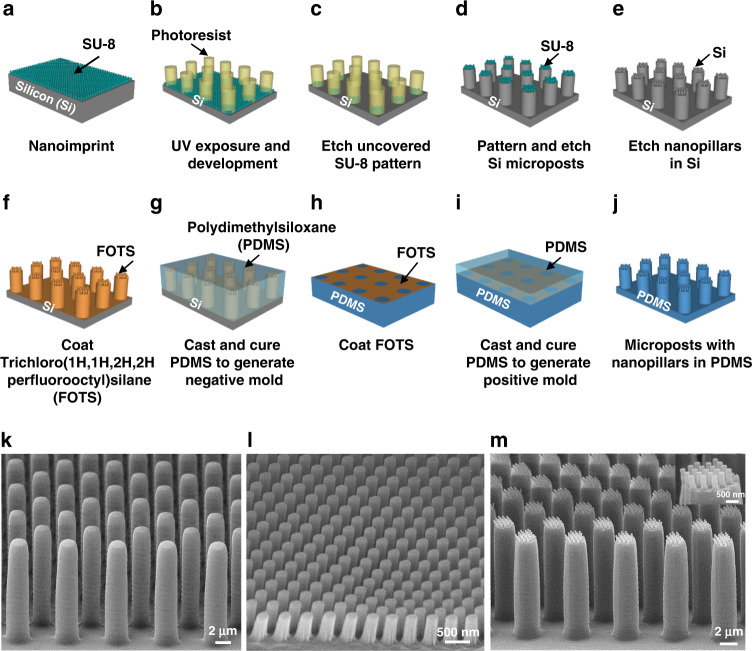
Schematics of fabrication technology for microposts with nanopillars. **a** SU-8 2000.5 is imprinted with simultaneous thermal and UV exposure. **b** UV lithography is used to pattern a nanopillar array. **c** Reactive ion etching in SF_6_/O_2_ to remove the uncovered SU-8 pattern. **d** Deep reactive ion etching (DRIE) of Si to form a micropost array and remove photoresist. **e** Removal of the residual layer of SU-8 nanopillars, DRIE of Si to form a nanopillar array and removal of SU-8 using plasma etching. **f**–**j** Double cast PDMS to generate the desired patterns. Trichloro(1H,1H,2H,2H perfluorooctyl)silane (FOTS) was used as an antisticking layer to promote easy demolding. Micrographs of (**k**) flat PDMS microposts, (**l**) surface with nanopillars, and (**m**) microposts with nanopillars.

Figure 1a–j show the schematics of the fabrication technology to pattern nanopillars on top of microposts. The silicon (Si) substrate was cleaned in acetone, isopropanol, and deionized (DI) water for 10 min and baked at 105 °C for 10 min. The hydrophilicity of the Si substrate was increased using O_2_ plasma with 20 sccm O_2_, 100 mTorr, and 100 W radio frequency (RF) power for 5 min. The SU-8 2000.5 polymer (Microchem, USA) was spin-coated on the Si substrate and baked at 65 and 95 °C for 2 min each. SU-8 nanopillars, which were 280 nm in diameter and 500 nm in height, were fabricated by nanoimprinting using an intermediate polymer stamp (IPS), as shown in Fig. 1a. The IPS was replicated from a nickel stamp with imprint conditions of 150 °C and 40 bar for 5 min and coated with trichloro(1H,1H,2H,2H-prefluorooctyl)silane (FOTS) to promote stamp separation after imprinting.

Next, a 4 µm-thick photoresist was spin-coated onto the SU-8 nanopillars and soft-baked at 95 °C for 15 min. After ultraviolet (UV) exposure through a photomask and development, the patterned micropost array was created, as shown in Fig. 1b. Reactive ion etching (RIE) (790 RIE system, Plasma-Thermal, USA) with a condition of 20/2 sccm SF_6_/O_2_, 10 mTorr, and 120 W RF power for 4 min was used to remove the nanopillars not covered by photoresist, as shown in Fig. 1c. The photoresist was also used as an etch mask to etch the 2.7 µm diameter and 12 µm height Si microposts with a deep reactive ion etching (DRIE) system (LPX ICP LE0729, SPTS, UK). The etch conditions were 138/11 sccm SF_6_/O_2_, 28 mTorr chamber pressure, 600 W coil power, and 14.8 W platen power at 13.56 MHz for 3.5 min, as shown in Fig. 1d. After removing the photoresist, the thin residual layer from the nanoimprint around the SU-8 nanopillars was removed by RIE with 20/2 sccm O_2_/SF_6_, 10 mTorr, and 120 W RF power for 2 min. Subsequently, the Si nanopillars on top of the microposts were formed by etching in the DRIE system with conditions of 70/35 sccm C_4_F_4_/SF_6_, 10 mTorr chamber pressure, 600 W coil power, and 10 W platen power for 1.2 min. Finally, the SU-8 etch mask was removed in the RIE system using an O_2_ plasma with 20 sccm O_2_, 10 mTorr, and 100 W RF power for 5 min, as shown in Fig. 1e. The Si mold was coated with FOTS as an antisticking layer, as shown in Fig. 1f. PDMS (base:crosslinker weight ratio = 10:1, Sylgard 184, Dow Corning, USA) was poured onto the Si mold coated with FOTS and degassed in a vacuum chamber at 10^−2^ bar for 2 h. The negative mold was generated after curing at 25 °C for 12 h, baking at 110 °C for 15 min and then coating with FOTS, as shown in Fig. 1g, h.

As shown in Fig. 1i, PDMS was spin-coated onto the negative mold and then degassed and baked as described earlier. Microposts with nanopillars were obtained after peeling the PDMS off the negative mold, curing at 25 °C for 12 h, and baking at 110 °C for 6 h, as shown in Fig. 1j. The PDMS micropost array was treated in a critical point dryer (EM CPD300, Leica, Germany) after being ultrasonicated in 100% ethanol (Sigma‒Aldrich, USA) for 1 min to prevent microposts from sticking to one another. Micrograph of flat PDMS microposts with 2.7 µm diameter, 12 µm height, and 3.3 µm spacing is shown in Fig. 1k. The surface with nanopillars of 220 nm diameter, 500 nm height, and 280 nm spacing is shown in Fig. 1l. In Fig. 1m, similar nanopillars were fabricated on top of the 12 µm-tall microposts. The microposts were in a hexagonal arrangement with a 3.3 µm edge-to-edge spacing between two adjacent microposts. The traction force sensitivity of the PDMS micropost array depended on the micropost design, including the height, diameter, and spacing of the microposts. Deep reactive ion etching technology was developed to form these tall microposts to achieve high sensitivity. The dry etching conditions were optimized to form tall microposts with small diameters. The spacing between the microposts was selected to ensure that the cells would stay on the top of the microposts while avoiding stiction with adjacent microposts^28^. In addition, the dimensions of the nanopillars were chosen so that they would have a significant influence on the cell migration behavior.

Furthermore, flat PDMS microposts were fabricated using fabrication technologies similar to those described above, as shown in Supplementary Fig. S1a–g. Microposts with silicon oxide or titanium oxide were formed by depositing silicon oxide or titanium oxide on top of the microposts using electron beam evaporation (ATS 500, HHV, UK), as shown in Supplementary Fig. S1h. The silicon oxide and titanium oxide were deposited using a 10 kV electron beam with deposition rates of 4.8 and 6.0 nm/min, respectively. The deposited oxides were directional since evaporation was carried out at a low pressure of 2 × 10^−5 ^Torr, corresponding to a mean free path of 402 cm. Since the distance between the source and the sample was 35 cm, this ensured that the oxides were directionally deposited only on the top and bottom of the microposts and not on the sidewalls^15,31^. In addition, the temperature of the sample stage during the 4 min evaporation was kept at 45 °C by water cooling when 20 nm thick silicon oxide or titanium oxide was evaporated on top of the microposts. Thus, the effect of temperature on the mechanical properties of the microposts was negligible. Subsequently, these micropost sensing platforms were adhered onto confocal dishes for further surface functionalization.

### Surface treatment of micropost sensing platforms

To keep the cells on top of the microposts and prevent cells from being trapped between microposts, all the micropost arrays were coated with fibronectin (FN) on top of the microposts and 0.2% Pluronic F-127 (Sigma‒Aldrich, USA) on the sidewalls of the microposts. Cells stayed on the FN-coated top surface of the microposts and did not migrate onto the micropost sidewalls because the Pluronic coating deterred the cells. To prepare such surface coating conditions for the micropost arrays, an FN-coated PDMS pad was first generated. A 1 × 1 × 0.5 cm^3^ PDMS pad (with a base: curing agent weight ratio of 20:1) was generated by curing on a 110 °C hotplate for 1.5 h after degassing in a 10^−2^ mbar vacuum chamber for 30 min. The PDMS pad was treated using an O_2_ plasma with 135 sccm O_2_, 15 sccm N_2_, 150 mTorr, and 25 W RF power within a Faraday cage for 15 s, and the water contact angle was measured to be 85° after the plasma treatment. Then, 25 µl FN (50 µg/ml, Sigma‒Aldrich, USA) was coated on the PDMS pad and kept at 4 °C for 4 h. The excess FN was removed by rinsing with DI water. After drying with N_2_, the PDMS pad with FN coating was used to transfer FN to the top of the microposts.

A plasma system (GIGAbatch 310 M, PVA TePla, Germany) was used to treat the micropost platform with O_2_ plasma using 135 sccm O_2_, 15 sccm N_2_, 150 mTorr, and 30 W RF power within a Faraday cage for 20 s. Subsequently, the FN-coated PDMS pad was placed in contact the PDMS micropost platform for 1 min 30 s, and FN was transferred to the tops of the microposts on the platform. Then, the platform was immersed in 100% ethanol for easy separation of the PDMS pad from the microposts, and the platform was ready for cell seeding.

The platform was immersed in 70% ethanol for disinfection and then rinsed twice with phosphate-buffered saline (PBS) in a biological safety cabinet. The micropost arrays were then labeled with a lipophilic dye (DiI, 5 mg/ml in distilled water, 1,10-dioleyl-3,3,30,30-tetramethylindocarbocyanine methanesulfonate, Invitrogen, USA) by submerging the platform in the dye at 25 °C for 90 min. The red dye made it easier to analyze the micropost displacement caused by the cell traction force using the fluorescence signal. After rinsing in PBS three times, the platform was immersed in 0.2% Pluronic F-127 (Sigma‒Aldrich, USA) and kept at 25 °C for 40 min to coat the micropost sidewalls with Pluronic. Finally, the platform was rinsed three times with PBS and submerged in PBS for cell culture and imaging.

### Cell culture and seeding

MC3T3-E1 osteoblastic cells were obtained from American Type Culture Collection (ATCC number CRL-2594) and maintained in high glucose Dulbecco’s modified eagle medium (DMEM, Invitrogen, USA) supplemented with 10% fetal bovine serum (FBS, Gibco, USA), antibiotic-antimycotic (100 units/ml of penicillin, 100 mg/ml of streptomycin, and 0.25 mg/ml of amphotericin B, Gibco, USA), and 2 mM alanyl-L-glutamine (Gibco, USA). Cells were incubated at 37 °C and 5% CO_2_, and the culture medium was changed every 2 days. After washing the PDMS substrates with 70% alcohol once and PBS twice, the MC3T3-E1 cells were seeded at a density of 3 × 10^4^ cells/cm^2^. The culture dish was placed in an incubator (37 °C, 5% CO_2_) for 6 h to allow the complete attachment of MC3T3-E1 cells onto the designed platforms.

### Cell migration trajectory, speed, and morphology analysis

The migration trajectory, speed, and morphology of MC3T3-E1 cells were analyzed using time-lapse images. After the cells attached and spread on the platform over 6 h, the medium was replaced by CO_2_-independent medium (Invitrogen 18045-088, USA), 10% FBS, and antibiotic-antimycotic medium supplemented with 2 mM alanyl-L-glutamine (Gibco, USA) for time-lapse imaging. Images were captured every 5 min for 16 h using an upright microscope (Eclipse Ni-E, Nikon, Japan). A 20× objective lens was used, and the cells were kept in an incubation chamber at 37 °C. The cell migration behaviors were analyzed using the Manual Tracking plugin of ImageJ software. Cells that were alive, not divided, and had no interaction with other cells during the 16 h imaging period were analyzed. All the data were from at least 3 independent assays. Statistical analysis was performed using one-way analysis of variance (ANOVA) and Tukey’s post hoc test.

### Traction force analysis using bent microposts

Time-lapse images were captured using a Nikon microscope at a time interval of 3 min for 16 h. A 50× objective lens was used. Bright-field images were captured to show cell positions, and fluorescent images of the stained microposts were captured to acquire the bending displacement of the microposts. The micropost sensing platforms consisted of micropost arrays with nanopillars or various coatings on top of the microposts. The presence of nanopillars or coatings did not change the spring constant of the microposts; hence, the traction force sensing remained effective with the micropost array. This was verified by simulations using a finite element analysis (FEA) suite (Multiphysics 5.4b, COMSOL, USA), as shown in Supplementary Fig. S2^28,32^. The results indicated that the fitted spring constant of 12.16 nN/μm for flat PDMS microposts, microposts with nanopillars, and microposts with oxide coatings was identical. The micropost displacement and spring constant were used to calculate the cell traction force using a custom-programmed graphical user interface in MATLAB (R2010b, The MathWorks, USA)^28^.

### Cell imaging using scanning electron microscopy

After time-lapse imaging, the cell culture medium in the dish was removed, and the platform was washed twice with 1% PBS for 5 min each time. Then, the cells were fixed with 4% paraformaldehyde for 15 min. After cell fixation, the platform was washed with 1% and 0.25% PBS for 5 min each, followed by rinsing twice in DI water for 10 min each time. Subsequently, cells were dehydrated for 5 min each time using a series of increasing ethanol concentrations (30%, 50%, 70%, 80%, 95%, and 100%). Cells were dried using a critical point dryer, in which CO_2_ was the transitional medium. A thin layer of gold was then sputter-coated on the platform using a thin film coater (Q150 coater, Quorum Technologies Ltd., UK). High-resolution images of the fixed cells were captured using a field emission scanning electron microscope (SEM) (SU5000 FE-SEM, Hitachi, Japan) with a 10 kV electron beam. These images were used to analyze the number and length of filopodia and long protrusions of cells. Typical filopodia were narrow with widths of 200–400 nm and lengths of 4–30 μm. Long protrusions had widths >400 nm, and protrusion length was defined as the distance between the edge of the cell membrane and the protrusion tip, which could be 5–50 μm long.

## Results and discussion

### Surface energy regulates cell responses

The physiochemical properties of the ECM could have strong effects on cell-ECM interactions^33^. In this study, PDMS was used as the base material for all surfaces, as shown in Supplementary Fig. S3a. A flat PDMS surface without any treatment was used as the control group. Three experimental groups with different surfaces were established. The first group focused on the effect of nanotopography and used PDMS with nanopillars fabricated on the surface. The second and third groups focused on the impact of the surface material and used PDMS with thin silicon oxide and titanium oxide layers deposited on the surfaces, respectively. The influence of the various surface conditions on cell migration behaviors was investigated. X-ray photoelectron spectroscopy (XPS) was used to study the chemical compositions of the oxides. Based on the XPS analysis, the silicon oxide was in the form of SiO_1.7_, and the titanium oxide was in the form of TiO_1.4_.

**Fig. 2:**
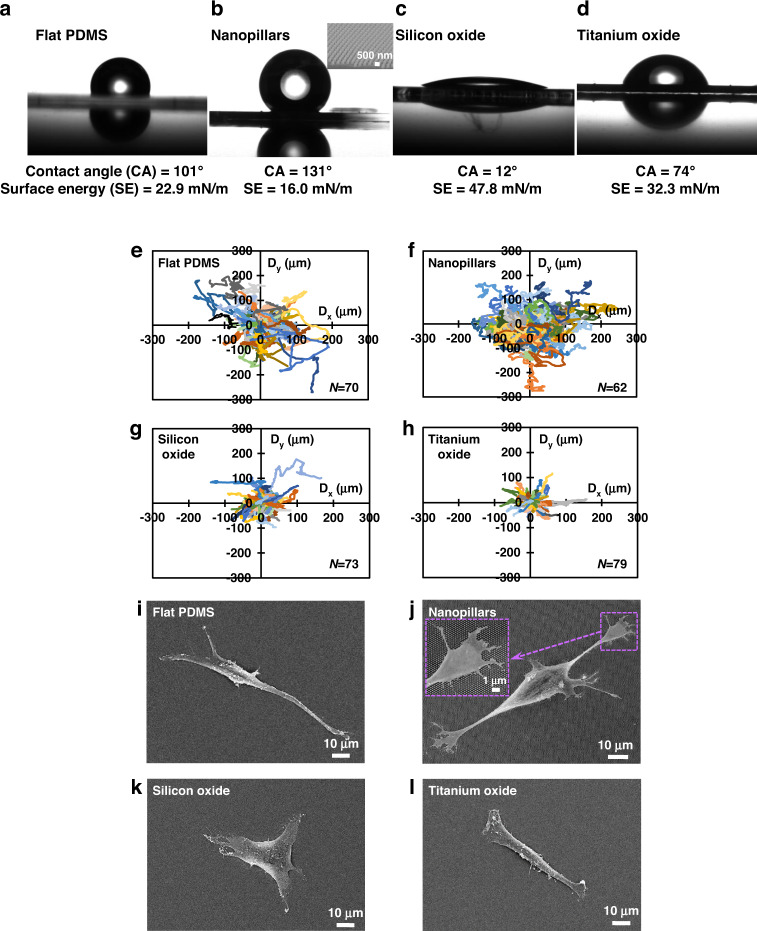
Dependence of cell migration behavior and cell morphology on surface energy. All surfaces without FN coating. Surface energy of **a** flat PDMS, **b** nanopillars, **c** silicon oxide, and **d** titanium oxide surfaces. Migration trajectories of MC3T3-E1 cells on **e** flat PDMS, **f** nanopillars, **g** silicon oxide, and **h** titanium oxide surfaces. Micrographs of cells on **i** flat PDMS, **j** nanopillars, **k** silicon oxide, and **l** titanium oxide surfaces.

The surface energy of an ECM or an engineered platform is related to its physical and chemical characteristics^34^. The water contact angle measurement was used to evaluate the surface energy of different surfaces on the microfabricated platforms. Figure 2 shows the water contact angle, migration trajectory, and cell morphology on different surfaces without FN coating. Surfaces with flat PDMS and nanopillars were hydrophobic (contact angle >90°) with larger water contact angles of 101° and 131°, respectively, corresponding to low surface energies of 22.9 and 16.0 mN/m, respectively, as shown in Fig. 2a, b. On the other hand, PDMS surfaces coated with 20 nm-thick silicon oxide or titanium oxide were hydrophilic (contact angle <90°); their water contact angles were 12° and 74°, respectively, and the corresponding surface energies were 47.8 and 32.3 mN/m, respectively, as shown in Fig. 2c, d. The surface energy of biomaterials can drive ligand topology and generate different focal adhesion signaling pathways between cells and substrates. These signals could modulate cytoskeletal tension and cause different cell responses^35^.

To observe the difference in cell behavior, videos were taken of MC3T3-E1 cell migration on different surfaces over 16 h with 5 min/frame (Supplementary movie SV1). As shown in Supplementary movies SV1 (a) and (b), some cells initially started in a circular shape. Once they contacted the PDMS or nanopillar surface, the cell membrane stretched out, the cell shape became elongated, and cell migration began. Changes in cell migration speed and morphology were dynamically monitored over 16 h. As shown in Fig. 2e–h, cells cultured on flat PDMS, nanopillar, silicon oxide, and titanium oxide surfaces had random migration trajectories. No guidance effect was found for cells on any of the platforms, and cells had similar migration speeds in the x- and y-directions, as shown in Supplementary Fig. S4. This is due to the lack of directional surface topography for cell migration guidance. However, cells that migrated on hydrophobic flat PDMS or nanopillar surfaces had longer paths and faster movements than those cells on hydrophilic oxide surfaces. The persistence lengths and kymographs of cell migration on different surfaces were analyzed to characterize the total travel distance of the migration path and the pace of the cell movement, as shown in Supplementary Figs. S5 and S6. Both the persistence lengths and the kymographs are consistent with the trajectories shown in Fig. 2e–h. Figure 2i–l show the distinctive morphologies of MC3T3-E1 cells on different surfaces. Cells cultured on hydrophobic flat PDMS or nanopillar surfaces possessed polarized morphologies, and the elongated cells had leading and trailing edges, indicating strong motile behavior, as shown in Fig. 2i, j. In particular, the presence of nanotopography can promote cell elongation, contraction, and ultimately cell migration. As shown in Fig. 2j, nanopillars established a discontinuous surface for cell adhesion. This made it easier for cells to attach to and detach from the surface during migration, leading to rapid cell movement^36^. Furthermore, deformation of the nanopillars was observed, suggesting that the nanopillars may be more conducive to transmitting cytoskeletal tension to the substrate^37^. On the surface with nanopillars, cells extended numerous filopodia in all directions, and filopodia extensions were more prominent around the advancing cell edge. However, cells grown on hydrophilic oxide surfaces showed less elongation and more spreading, as shown in Fig. 2k, l. One plausible explanation for these differences is related to the surface energy modulation of the distribution and deposition of adsorbed proteins^38^. These results indicate that surface energy could be a critical factor affecting cell migration behaviors and cell shapes.

### Cell migration and cell morphology on different surfaces

**Fig. 3:**
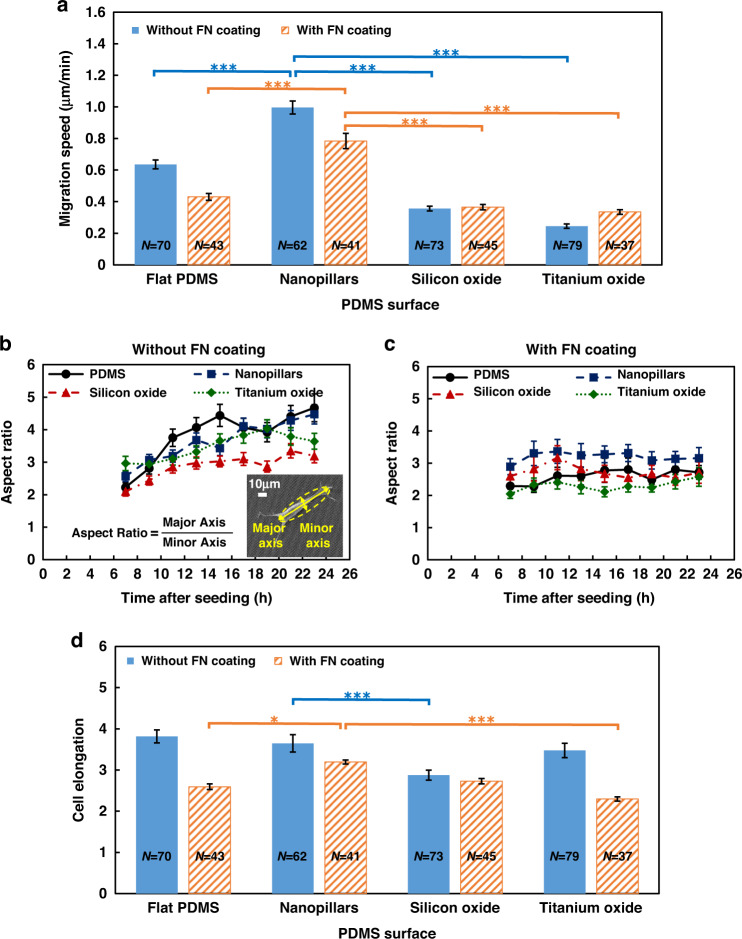
Cell migration speed, aspect ratio, and elongation of MC3T3-E1 cells on different surfaces with and without FN coating. **a** MC3T3-E1 cell migration speed on flat PDMS, nanopillar, silicon oxide, and titanium oxide surfaces with and without FN coating. Changes in the cell aspect ratio over 16 h on different surfaces **b** without and **c** with FN coating. **d** Cell elongation on different surfaces with and without FN coating. One-way ANOVA and Tukey’s post hoc test, *p < 0.05 and ***p < 0.001.

Figure 3 shows the migration speed, aspect ratio, and elongation of MC3T3-E1 cells on different surfaces with and without FN coating. The migration speeds of cells on flat PDMS, nanopillar, silicon oxide, and titanium oxide surfaces without FN were 0.64, 0.99, 0.36, and 0.25 μm/min, respectively, as shown in Fig. 3a. Cells on nanopillars had the highest migration speed compared to other surfaces due to the difficulty of forming focal adhesion sites on hydrophobic surfaces, resulting in weak adhesion and hence faster migration speed^39^. In contrast, hydrophilic surfaces promoted stronger cell adhesion and resulted in slower cell migration speed. Moderately hydrophilic surfaces with contact angles between 60° and 80° have been shown to have the highest cell adhesion^23,40,41^. On the titanium oxide surface, the water contact angle was 74°, corresponding to the highest cell adhesion and the slowest migration speed^17^.

With the FN coating, the surface energy on the flat PDMS, nanopillar, and titanium oxide surfaces increased, which is reflected by the decreased water contact angles, as shown in Supplementary Fig. S7. As a result, the cell migration speed on flat PDMS and nanopillars decreased after FN coating due to the enhanced cell adhesion. Interestingly, cells on nanopillars coated with FN still showed a higher migration speed at 0.78 μm/min than all the other FN-coated surfaces, although the 82° water contact angle indicated that the cell adhesion should be high enough to make the cells less mobile. Thus, it is suggested that the FN coating partially overrides the effect of the substrate material.

Changes in the cell aspect ratio over 16 h on different surfaces without FN are shown in Fig. 3b. The cell aspect ratio is defined as the ratio between the major and minor axes when cells are fitted to an ellipse^10^. Initially, cells on the flat PDMS and nanopillar surfaces had a more rounded shape, and the aspect ratio was small because of the unstable attachment to the hydrophobic surface^23^. Cells formed a stable attachment to the PDMS and nanopillar surfaces over time, and thus the aspect ratio increased with time. However, there was more frequent cytoskeletal rearrangements in cells on nanopillars than in cells on flat PDMS surfaces, which corresponded to more rapid cell movements, as shown in Supplementary movie SV1(b)^42^. In addition, the aspect ratio for cells on the silicon oxide surface remained the smallest for 16 h. With FN coating, the protein layer facilitated cell spreading in multiple directions, leading to a more rounded cell shape. The cell morphology was also more stable over 16 h, as shown in Fig. 3c. The nanopillar surface led to a slightly larger cell aspect ratio than the other surfaces after FN coating.

Furthermore, cell elongation was obtained by averaging the aspect ratio over 16 h, as shown in Fig. 3d. Before FN coating, the elongation of cells on nanopillars was similar to that of cells on flat PDMS and titanium oxide surfaces. However, since the surface energy of the silicon oxide surface was the highest, cells could more easily adhere to the surface in multiple directions, resulting in a more rounded cell shape. After FN coating, cells on nanopillars still had the largest cell elongation among all the tested surfaces. This provides further evidence that nanotopography has a more profound effect on cell migration behavior.

These results may be related to surface energy-driven protein adsorption^43,44^. Focal adhesions are formed by the binding of integrin receptors to FN adsorbed on the surface, constituting a mechanotransduction link between the cytoskeleton and the ECM. This can mediate cell tension to cause the cell to adapt its shape and exhibit complex mechanocoupling responses to achieve cell migration^9^. The formation of focal adhesions is a prerequisite for the generation of cell traction force. The cell adhesion area, protein adsorption, and fibrillar adhesion are changed and thus influence the complex force from the cell that acts on the ECM surface. Additionally, nanotopography influences protein adsorption, and thus cell migration, by surface energy and also profoundly affects the mechanotransduction between the cell and the substrate with its specific physical structure.

### Cell traction force exerted on microposts with nanopillars or oxide coatings

Micropost sensing arrays were used to measure the cell traction force on four surface conditions by fabricating PDMS nanopillar arrays, applying flat silicon oxide, or applying flat titanium oxide on top of PDMS microposts, as shown in Supplementary Fig. S3b. All the microposts with various surfaces were coated with FN on top and Pluronic on the sidewalls of the microposts to keep the cells on top of the microposts for the cell traction force study. Supplementary Fig. S8 shows that the cell migration speeds on flat PDMS surfaces and on flat PDMS microposts were not significantly different. Similarly, there was no significant difference between the nanopillar surface and microposts with nanopillars. Since cell migration on a large surface area and on the top surfaces of microposts was similar, it was concluded that the micropost arrays could be applied to sense the cell traction force.

**Fig. 4:**
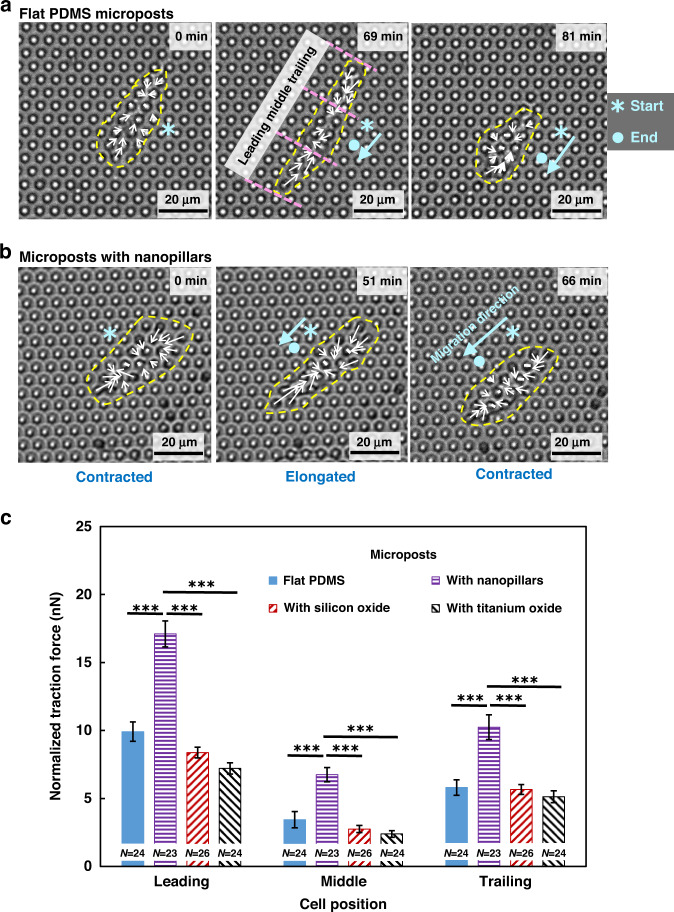
Cell traction force exerted on microposts with various surfaces. Traction force development for MC3T3-E1 cell migration on PDMS microposts with **a** flat PDMS and **b** with nanopillar surfaces. **c** Normalized traction force in the leading, middle, and trailing regions of cells on microposts with various surface conditions. The tops of microposts were coated with FN and the sidewalls were coated with Pluronic. The yellow dashed line indicates the cell contour. The starting and ending positions are indicated by asterisks and dots in the micrographs, respectively. The white arrows indicate the traction force on the microposts. The length of the white arrow represents the magnitude of the traction force. Cell migration direction is marked by a blue arrow, representing the movement of the cell centroid during a single migration cycle. As the cell traction force varies from the leading to trailing region during migration, each cell is divided into three distinct isometric regions along its migration axis, including the leading, middle, and trailing regions. One-way ANOVA and Tukey’s post hoc test, ***p < 0.001.

Figure 4 shows the development of traction force for MC3T3-E1 cell migration on PDMS microposts and on PDMS microposts with nanopillars, silicon oxide coating, or titanium oxide coating. Figure 4a shows the changes in cell morphology and traction force for MC3T3-E1 cells during forward migration on flat PDMS microposts. The measured traction force was directed toward the cell center, with a higher force acting around the cell periphery and a lower force acting at the cell center. During migration, the cell elongated by protruding the leading and trailing regions. The corresponding traction force increased gradually, and a higher force was found near the leading region compared to the trailing region of the cell. There was very little forward movement of the cell when it was elongated. Then, the trailing region detached from the microposts, and the traction force dropped in both the leading and trailing regions. As the trailing region retracted, the cell moved forward and started the migration cycle again. This cyclic behavior repeated itself during cell migration and is consistent with previous findings^28,29^.

As shown in Fig. 4b, cells that migrated on microposts with nanopillars had similar cyclic behavior but showed greater amplitudes of traction force in all three cell regions and shorter cycle times. Meanwhile, cells on microposts with silicon oxide or titanium oxide also had similar cyclic behavior. However, cells migrated with lower amplitudes of traction force and longer cycle time. These results indicate that cell migration was cyclic, and a shorter cycle time and higher migration speed corresponded to cell migration on the surface with a larger traction force. In addition, elongated cells generated a larger traction force compared to contracted cells, which may be due to the greater actin cytoskeleton tension during cell elongation. Being able to assess the traction force distribution of cells during migration will provide a better understanding of the cell migration mechanisms.

Figure 4c shows the normalized net traction force in the leading, middle, and trailing regions when cells were elongated. For cells seeded on flat PDMS microposts, the traction force exerted in the leading region was 9.9 ± 0.7 nN, >3.4 ± 0.6 nN in the middle region and 5.8 ± 0.6 nN in the trailing region. The trend of traction force generally agrees with previous reports^28,29^. In comparison, the traction force of the leading, middle, and trailing regions for cells seeded on microposts with nanopillars was 17.1 ± 1.0, 6.8 ± 0.5, and 10.2 ± 0.9 nN, respectively, which was larger than that on flat PDMS microposts. For cells on the silicon oxide surface, the traction force was smaller in the leading (8.4 ± 0.4 nN), middle (2.8 ± 0.3 nN), and trailing (5.7 ± 0.4 nN) regions when compared with the traction forces of cells on the flat PDMS surface. For cells on the titanium oxide surface, the traction force in the leading, middle, and trailing regions was 7.2 ± 0.4, 2.4 ± 0.3, and 5.1 ± 0.4 nN, respectively, which are smaller than the traction forces of cells on the silicon oxide surface.

In general, cells on all surfaces had a large force imbalance from the leading to trailing regions. The traction force of the leading region was the largest, and that of the middle region was the smallest. Furthermore, the force in the leading region was greater than that in the trailing region when cells were moving forward. This may be because there were more filopodia in the leading region than in the trailing region, which was related to the ligand adhesion that formed a larger fibrillar adhesion force. Cells on various surfaces had traction forces with different magnitudes, which corresponded to the related cell migration speed. Cells on the nanopillars had the largest traction force, which corresponded to the fastest migration speed. With the introduction of nanotopography, nanopillars could easily absorb proteins, which facilitated the transmission of traction force to the substrate^45^. Additionally, nanopillars could promote the formation and extension of filopodia and long protrusions; therefore, the fibrillar adhesion force between the cells and the substrate could be enhanced. Hence, it can be inferred that a strong fibrillar adhesion force as well as good transmission of traction force result in a large cell traction force and high migration speed on the surface with nanopillars^46^.

### Cells that migrated on microposts with nanopillars had more filopodia and long protrusions

**Fig. 5:**
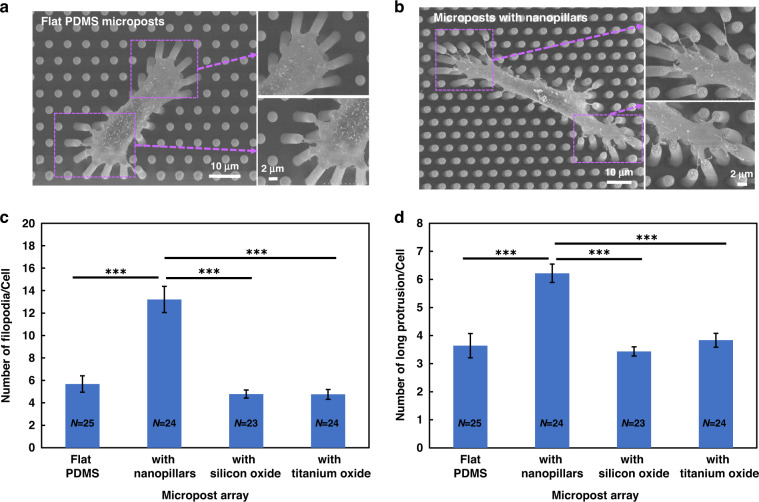
Morphologies, filopodia, and long protrusions of MC3T3-E1 cells on different platforms. Micrographs of MC3T3-E1 cells on microposts with **a** flat PDMS and **b** nanopillar surfaces. Number of **c** filopodia and **d** long protrusions per cell from scanning electron micrographs. Microposts were coated with FN on top and Pluronic on sidewalls. One-way ANOVA and Tukey’s post hoc test, ***p < 0.001.

Figure 5 shows the distinctive morphologies of MC3T3-E1 cells on different platforms and the quantitative analysis of filopodia and long protrusions. Cells attached to the tops of microposts due to the FN selectively coated on the tops and Pluronic F-127 on the sidewalls of microposts^32^. As cells migrated, the microposts bent under the cell traction force, and the displacement of the microposts was used to quantify the traction force exerted by the cells. As shown in Fig. 5a, cells on flat PDMS microposts bent the microposts toward the cell center due to cell traction force, similar to cells migrating on top of microposts with nanopillars, as shown in Fig. 5b. In addition, microposts with silicon oxide or titanium oxide bent in a similar manner, as shown in Fig. 5a, b. However, cells on microposts with nanopillars had more filopodia and long protrusions, which was consistent with our previous findings that nanostructures promote the generation of filopodia and long protrusions^19^.

High-resolution SEM was utilized to quantify the number and length of filopodia and long protrusions on different platforms, as shown in Fig. 5c, d and Supplementary Fig. S9a, b. The MC3T3-E1 cells on flat PDMS microposts typically had 6 filopodia/cell and 4 long protrusions/cell. Cells on microposts with silicon oxide and titanium oxide typically had 3 and 4 long protrusions, respectively, and the same number of 5 filopodia/cell. For cells on microposts with nanopillars, the numbers of filopodia/cell and long protrusions/cell were 13 and 6, respectively. More filopodia and long protrusions were formed on microposts with nanopillars on top, which resulted in a larger cell traction force. However, there was no significant difference in the length of filopodia or long protrusions, as shown in Supplementary Fig. S9a, b. Since the microposts were separated from one another, the discontinuous gaps between the microposts may limit the extensions of filopodia and long protrusions. Having more filopodia and long protrusions extended from the cell membrane resulted in a larger traction force and thus gave rise to the higher migration speed of MC3T3-E1 cells on nanopillars^19^. These results agreed with previous observations that more actin-rich protrusions from cell edges generated larger contractile forces during cell migration^47^.

### Dynamic traction force monitored on various surfaces

**Fig. 6:**
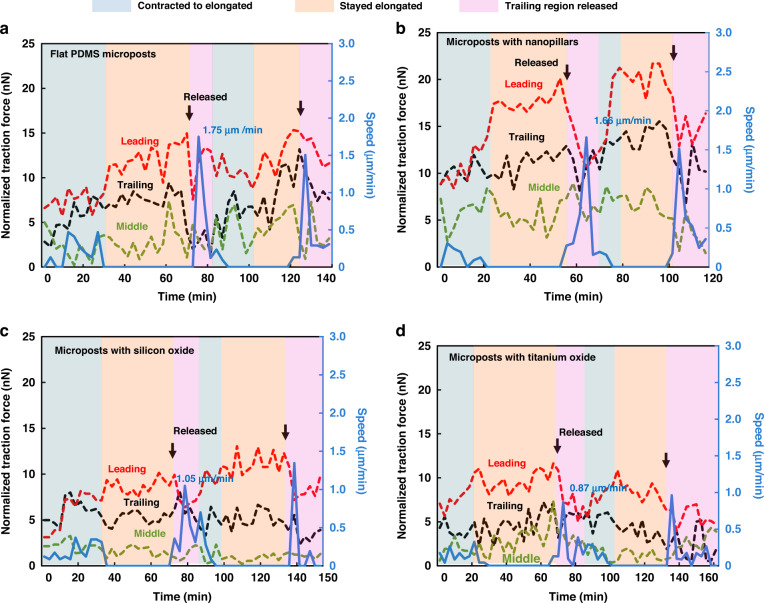
Dynamic traction force of MC3T3-E1 cells on different surfaces. Normalized cell traction force and speed as a function of time for MC3T3-E1 cells migrating on PDMS microposts with **a** flat PDMS, **b** nanopillars, **c** silicon oxide, and **d** titanium oxide. Microposts were coated with FN on top and Pluronic on the sidewalls. The maximum instantaneous speed is indicated.

During directional migration, cells undergo cyclic dynamic changes, during which the distribution of cell traction force from the leading front to the trailing edge can affect cell migration^48^. Thus, the measurement of cell traction force development over time from the leading to the trailing regions during cell migration is essential to provide insights into cell migration dynamics. Using the force vectors measured by the microposts and normalizing the force by the number of microposts covered by a cell, the cell traction force in the three regions and cell migration speed were analyzed as a function of time, as shown in Fig. 6.

Figure 6a shows that cells migrating on flat PDMS microposts contracted initially at 0 min with a relatively smaller traction force for all regions. Over time, the cells became elongated with increased traction force. The traction force of the leading region was larger than that of the trailing region, and the traction force of the middle region was the lowest. The trailing region started to release at 69 min as the traction force dropped in both the leading and trailing regions. After the trailing region was completely disconnected from the microposts at 81 min, the cells contracted, and the next migration cycle began. The cyclic changes in the traction force and cell morphology agree with our previous findings^28^. As the cell shape changed from contraction to elongation, lamellipodia protruded over time and formed leading, middle, and trailing regions. The traction force increased gradually when the cell was elongated, but the cell barely moved. The cell speed increased significantly after the adhesive sites in the trailing region were released, and the cell moved forward. Then, the next cell migration cycle started, and the change in speed was repeated.

For cells migrating on microposts with nanopillars, silicon oxide, and titanium oxide, the cyclic changes of the time-dependent traction force were similar to those of cells on flat PDMS microposts, as shown in Fig. 6b–d. Compared with the other three surfaces, the surface with nanopillars led to a larger traction force, probably related to the rapid skeletal reorganization of the cells on the nanostructures, as mentioned above. With the release of the trailing region, the cell speed increased as the cell moved ahead. A cytoskeleton reorganization cycle was defined as the time needed between two consecutive maximum instantaneous speeds. The comparison of dynamic traction force on different surfaces is summarized in Table 1. The cycle times of the cells on microposts with flat PDMS, nanopillar, silicon oxide, and titanium oxide surfaces were 54, 42, 63, and 66 min, respectively. Cells on the microposts with nanopillars had the shortest cycle time.

**Table 1:** Comparison of the dynamic traction force on different surfaces

Surface conditions of the microposts	Cycle time (min)	Instantaneous migration speed (µm/min)	Corresponding traction force (nN)
Flat PDMS	54	1.75	12.08
Nanopillars	42	1.66	11.62
Silicon oxide	63	1.05	7.15
Titanium oxide	66	0.87	6.98

Furthermore, the maximum instantaneous speeds were 1.75, 1.66, 1.05, and 0.87 μm/min. The corresponding traction forces were 12.08, 11.62, 7.15, and 6.98 nN, respectively. To a certain extent, the maximum instantaneous speed was higher for a larger traction force. In addition, the dynamic cell traction force on microposts with nanopillars was the largest throughout the cell migration cycle compared to the other three surfaces. The larger dynamic traction force also correlated with a higher migration speed. It is evident that dynamic traction force is a function of surface topography and coating that can strongly influence cell migration. Using micropost arrays to monitor cell traction force, cells on various surfaces showed similar time-dependent traction force changes during migration. However, the magnitude of the cell traction force depended on the surface topography and coating on the micropost surface. These results will provide important information related to the interactions between osteoblastic cells and ECM.

## Conclusions

In this study, various surface topographies and coatings, including flat PDMS, nanopillar, silicon oxide, and titanium oxide surfaces, were developed to study their effects on cell migration behaviors. Surfaces with flat PDMS or nanopillars were hydrophobic, while surfaces coated with silicon oxide or titanium oxide were hydrophilic. The results were related to cell migration speed and changes in cell morphology, including cell aspect ratio and elongation, due to the changes in surface energy. Cells on hydrophobic surfaces with lower surface energy showed higher migration speed and greater cell elongation than those on hydrophilic surfaces with higher surface energy. Cells migrated fastest (0.99 µm/min) on the nanopillar surface and slowest (0.25 µm/min) on the titanium oxide surface. Furthermore, the effects of the protein coating on cell migration behaviors were analyzed. The cell migration speed on nanopillars with FN coating was 0.78 µm/min, which was still significantly different from that of cells on other surfaces with FN coating. The different migration speeds may be related to the dependence of FN adsorption on different surfaces that have different surface energies.

PDMS micropost sensing platforms with various topographies and coatings were used to study cell traction force over time to correlate traction force development with surface conditions and migration behaviors. Cells that migrated on microposts with nanopillars had cyclic behaviors and generated the largest traction force on the microposts compared to cells migrating on flat PDMS microposts. For cells elongated on microposts with nanopillars, the traction force was larger in the leading (17.1 nN), middle (6.8 nN), and trailing (10.2 nN) regions compared to cells on the flat PDMS surface (traction forces of 9.9, 3.4, and 5.8 nN in the leading, middle, and trailing regions, respectively). More filopodia/cell and long protrusions/cell were observed for the cells on microposts with nanopillars compared to those on flat PDMS microposts, corresponding to the higher traction force and faster migration speed. To the best of our knowledge, this is the first study to analyze the dynamic cell traction force on different surfaces using micropost arrays. These results provide a better understanding of cell migration on surfaces with various topographies and coatings, which could be valuable in developing smart bionic platforms for regenerative medicine.

## Supplementary information

Supplementary Figures

Supplementary Movie
