# Supplementary material for: Effects of nanopillars and surface coating on dynamic traction force
Source: Microsyst Nanoeng. 2023 Jan 5;9:6. doi: 10.1038/s41378-022-00473-0 (PMC9814462; doi:10.1038/s41378-022-00473-0)
Supplement: Supplementary file 1 — Supplementary Figures [file 41378_2022_473_MOESM1_ESM.pdf]

Electronic Supplementary Information (ESI)

Effects of Nanopillars and Surface Coating on Dynamic Traction Force

Yijun Cheng[1,2] and Stella W. Pang[1,2]*

[1]Department of Electrical Engineering, City University of Hong Kong, Kowloon, Hong Kong

[2]Centre for Biosystems, Neuroscience, and Nanotechnology, City University of Hong Kong, Kowloon, Hong Kong

*Corresponding Author: pang@cityu.edu.hk

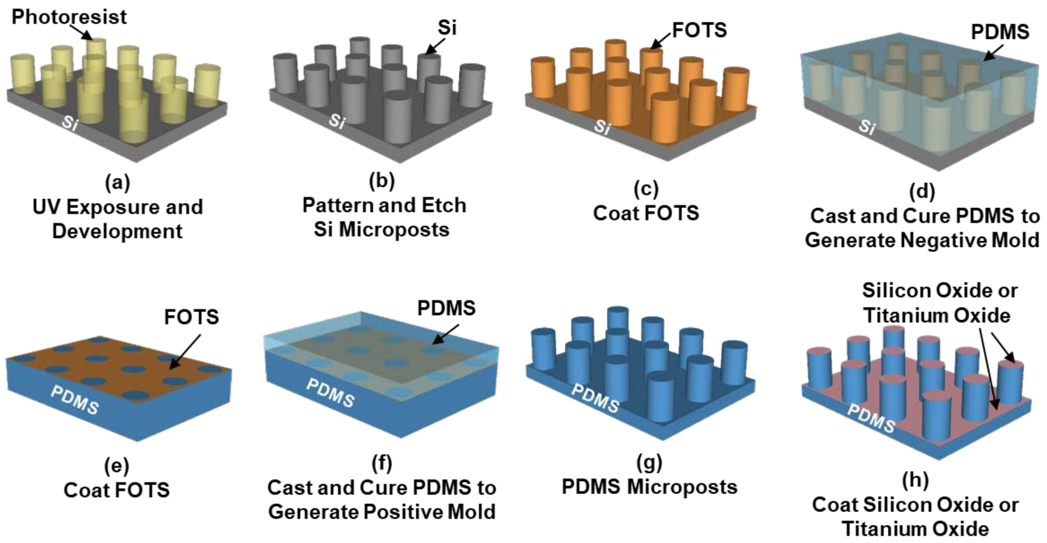

Supplementary Figure S1. Schematics of fabrication technology for microposts with oxide coating.

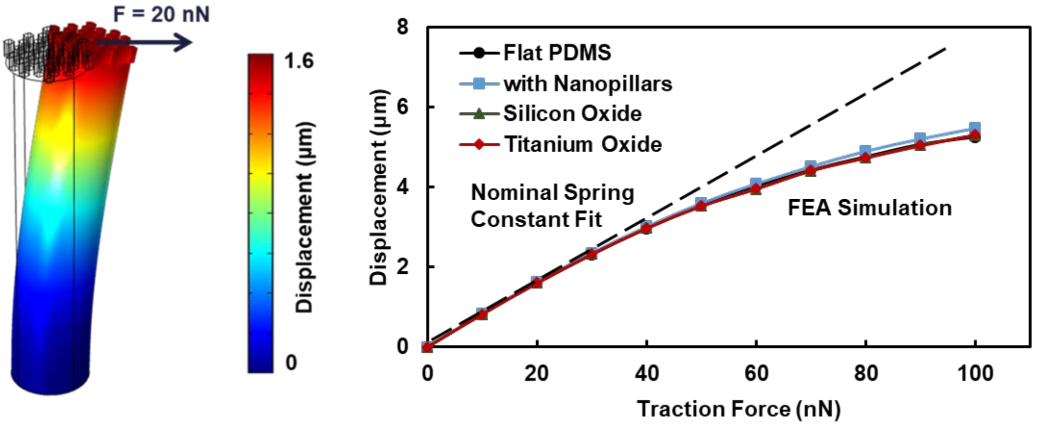

Supplementary Figure S2. Finite element analysis showing displacement as function of traction force of elastic micropost with diameter of 2.7 μm and height of 12 μm with flat PDMS, with nanopillars of 220 nm diameter, 500 nm height, and 280 nm spacing, and with 20 nm thick silicon oxide or titanium oxide on top surfaces.

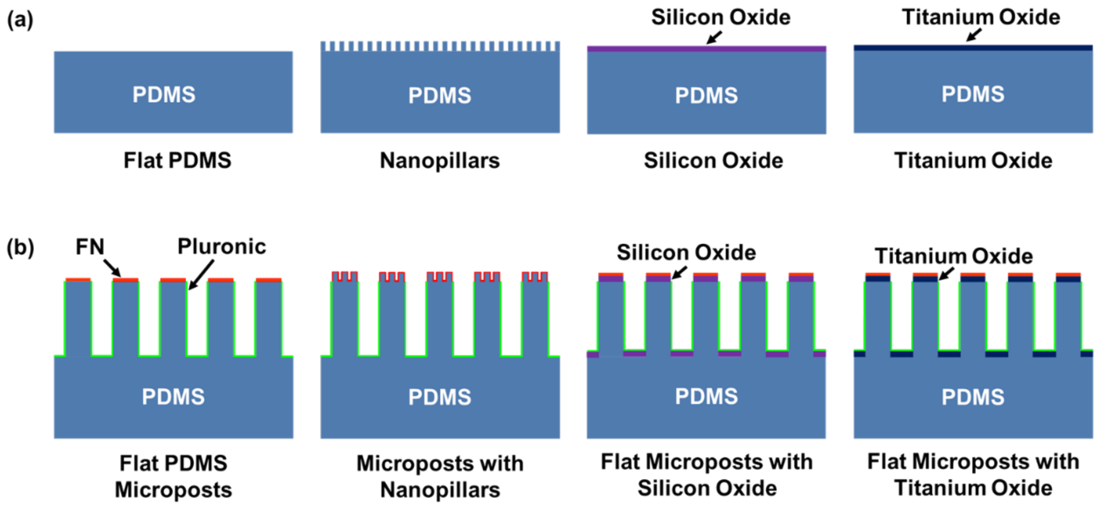

Supplementary Figure S3. Schematic diagrams of (a) flat PDMS, nanopillar, silicon oxide, and titanium oxide surfaces, and (b) flat PDMS microposts, microposts with nanopillars, and flat PDMS microposts with silicon oxide or titanium oxide.

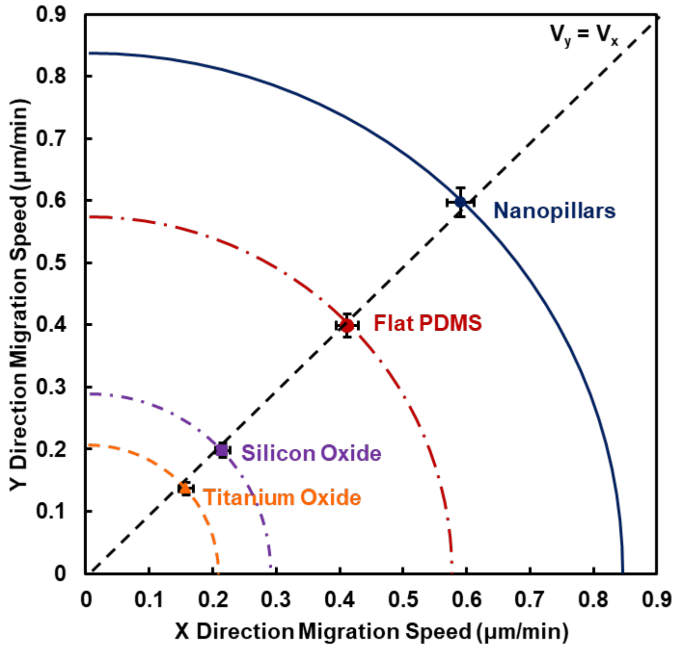

Supplementary Figure S4. All surfaces without FN coating. Cell migration speed in x and y directions of MC3T3-E1 cell on various platforms over 16 h.

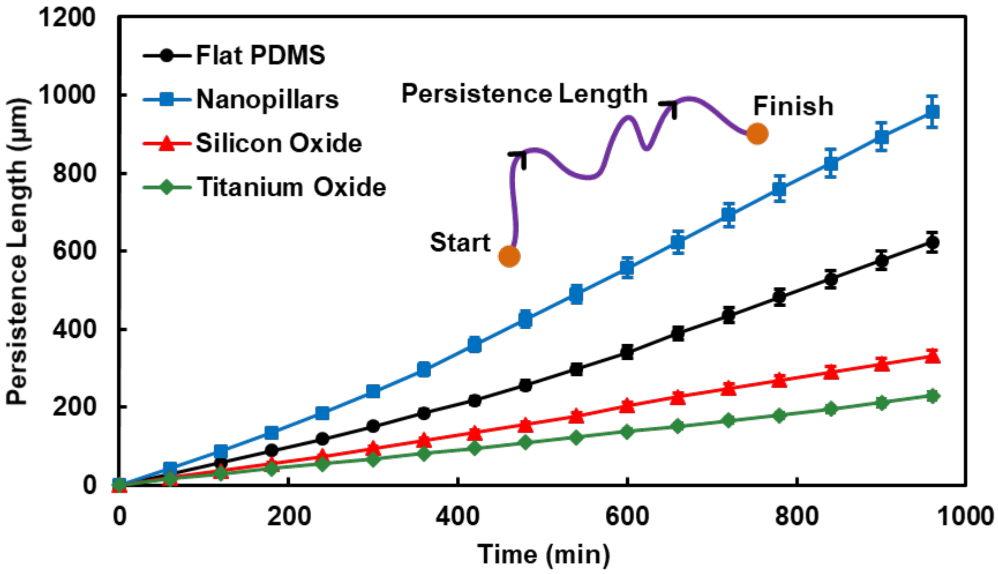

Supplementary Figure S5. Persistence length of cell migration on PDMS surfaces that were flat, with nanopillars, and with silicon oxide or titanium oxide coating.

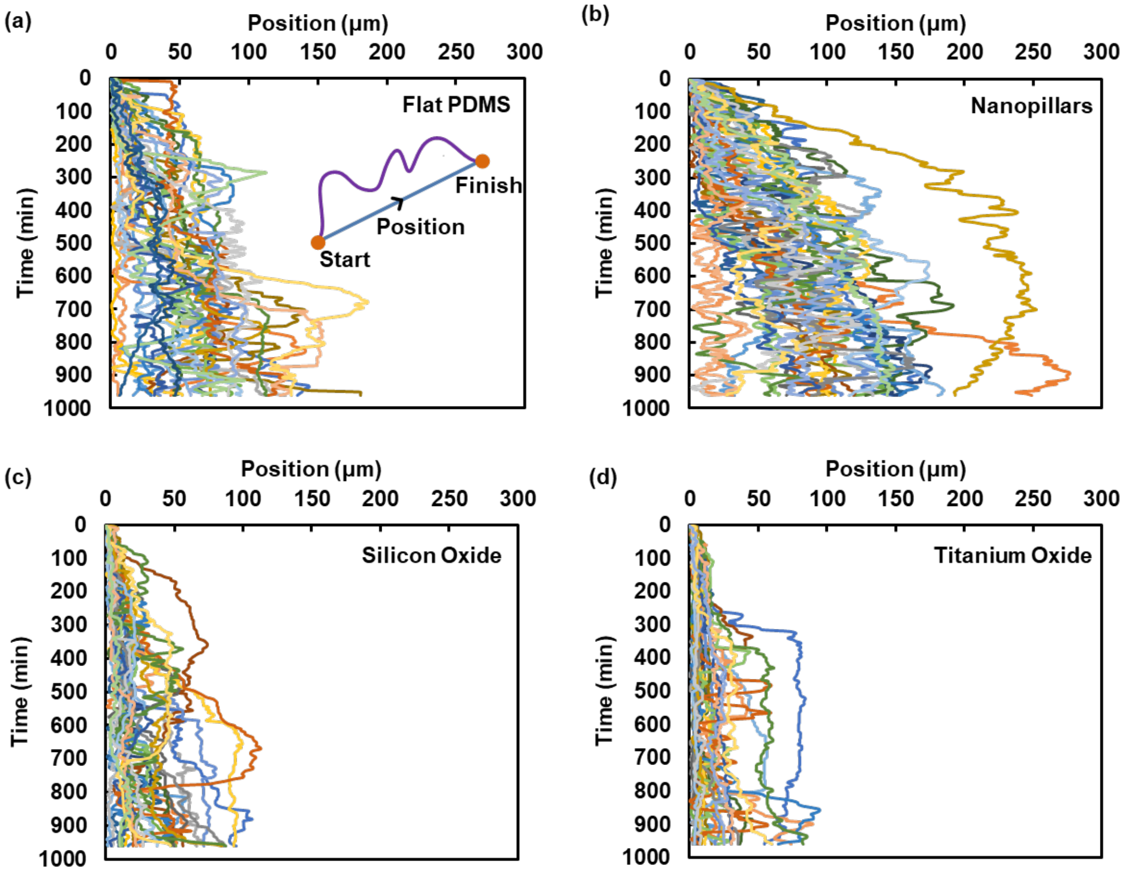

Supplementary Figure S6. Kymograph of cell migration on PDMS surfaces that were flat, with nanopillars, and with silicon oxide or titanium oxide coating.

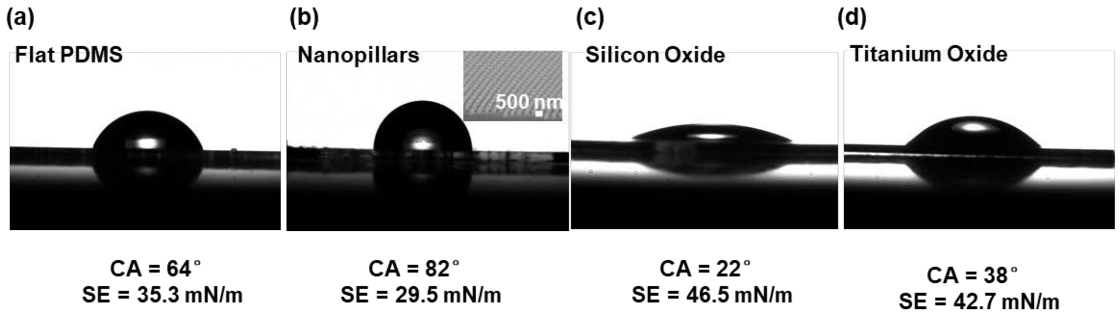

Supplementary Figure S7. Surface energy of (a) flat PDMS, (b) nanopillars, (c) silicon oxide, and (d) titanium oxide. All surfaces were coated with FN.

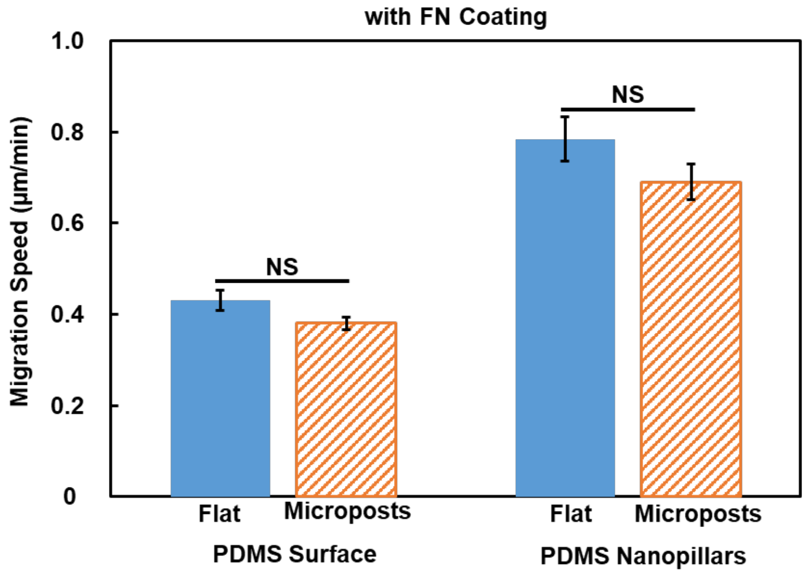

Supplementary Figure S8. Cell migration speed on flat PDMS surface, microposts with flat PDMS, nanopillar surface, and microposts with nanopillars. All top surfaces were coated with FN. One-way ANOVA and Tukey's post hoc test, NS – not significant.

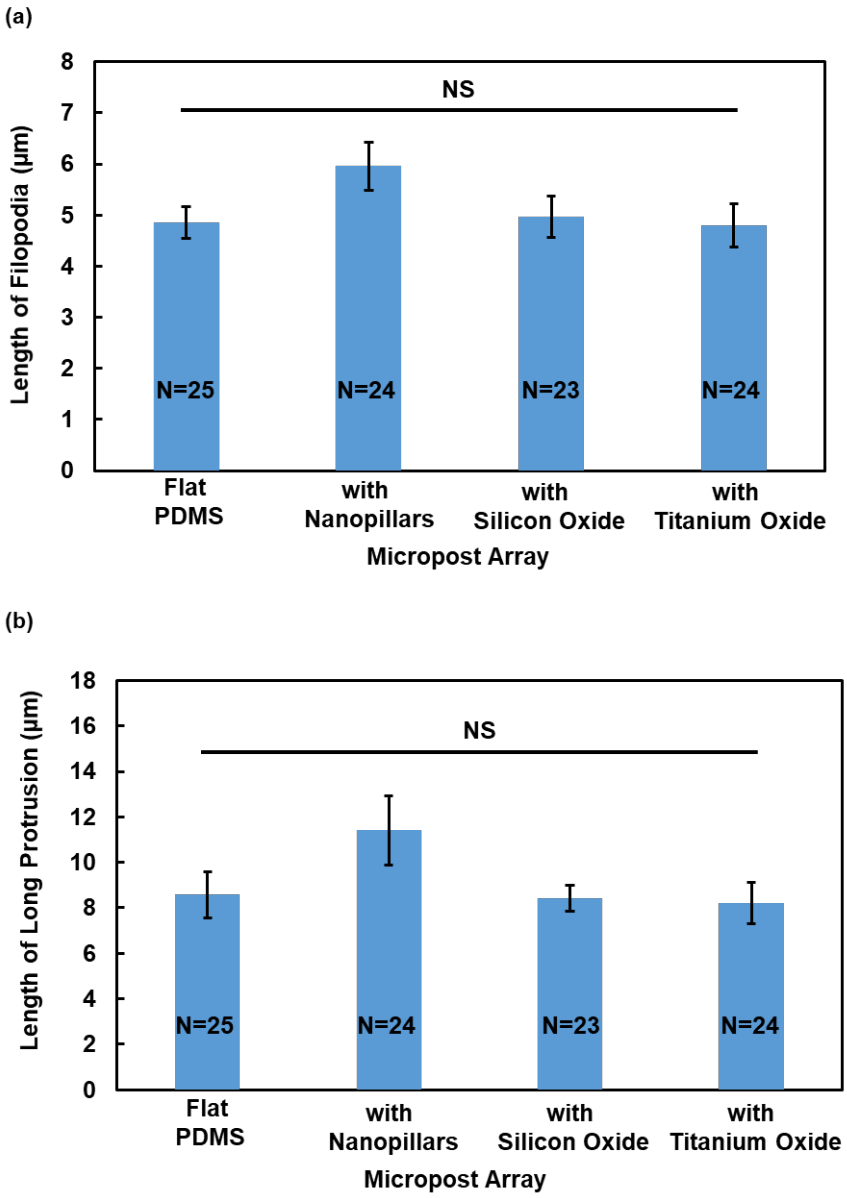

Supplementary Figure S9. Length of (a) filopodia and (b) long protrusions for cells on microposts with various surfaces. Microposts were coated with FN on top and Pluronic on sidewalls. One-way ANOVA and Tukey's post hoc test, NS – not significant.
